# Guided bone regeneration improves defect fill and reconstructive outcomes in 3-wall peri-implantitis defects

**DOI:** 10.1038/s41432-024-01073-9

**Published:** 2024-10-14

**Authors:** Kelvin I. Afrashtehfar, Hayam A. Alfallaj, Eduardo Fernandez, Souheil Hussaini

**Affiliations:** 1 Department of Reconstructive Dentistry and Gerodontology, School of Dental Medicine, University of Bern, Bern, Switzerland; 2 Private Practice, Abu Dhabi City, UAE; 3 Department of Restorative and Prosthetic Dental Sciences, College of Dentistry, King Saud Bin Abdulaziz University for Health Sciences, Riyadh, Saudi Arabia; 4 King Abdullah International Medical Research Center (KAIMRC), Riyadh, Saudi Arabia; 5 Restorative Dentistry Department, University of Chile, Santiago, Chile; 6 Instituto de Ciencias Biomédicas, Universidad Autónoma de Chile, Santiago, Chile; 7 Oral Implantology Research Institute, Dubai, UAE; 8 Private Practice, Dubai, UAE

**Keywords:** Peri-implantitis, Dental implants, Dental implants

## Abstract

**Design:**

This multi-center, randomized clinical trial compared the long-term outcomes of guided bone regeneration (GBR) with open flap debridement (OFD) in treating peri-implantitis-related bony defects with ≥3 osseous walls over 36 months. The study aimed to evaluate the healing potential of GBR using a deproteinized bovine bone mineral (DBBM) graft and native bilayer collagen membrane (NBCM) compared to OFD without the use of graft materials.

**Case selection:**

Sixty-six individuals diagnosed with peri-implantitis were randomly assigned to either GBR (34 patients) or OFD (32 patients). The OFD group served as the control, where inflamed tissue was removed and the implant surface decontaminated using 3% hydrogen peroxide, but no bone graft was placed. The GBR group received DBBM and NBCM for defect reconstruction. Radiographic defect fill (RDF), probing pocket depth (PPD), bleeding on probing (BOP), suppuration (SUP), mucosal recession (MREC), and patient-reported outcomes (PROs) were assessed over the study duration. Post-surgical care included azithromycin, ibuprofen, and chlorhexidine rinses.

**Study timeline:**

The study involved baseline assessments, surgical interventions, and follow-ups at 6, 12, and 36 months. Supportive peri-implant therapy was provided every 3 months during the additional 24-month follow-up.

**Data analysis:**

Primary outcome was RDF at 36 months. Secondary outcomes included PPD, BOP, SUP, MREC, and PROs. Descriptive statistics and ANCOVA models were used for analysis.

**Results:**

At 36 months, GBR resulted in a mean RDF of 2.13  ±  1.26 mm, compared to 1.64  ±  1.54 mm with OFD (p  =  .18). No significant differences were found in PPD, BOP, SUP, REC, or PROs between the groups. Treatment success (defined as no additional bone loss, PPD ≤ 5 mm, no BOP, and no SUP) was achieved in 46.2% of GBR cases and 20% of OFD cases (p = 0.053).

**Conclusions:**

GBR provided improved short-term defect fill and higher treatment success compared to OFD, although the differences were not statistically significant. Both procedures maintained clinical parameters over 36 months, with similar patient satisfaction (PROs) observed for GBR and OFD. The adjunct use of DBBM and NBCM may offer clinical benefits for peri-implantitis cases with specific bony defect morphology.

## A Commentary on

**Renvert S, Giovannoli J-L, Rinke S**

The efficacy of reconstructive therapy in the surgical management of peri-implantitis: a 3-year follow-up of a randomized clinical trial. *J Clin Periodontol* 2024; 10.1111/jcpe.14049

**GRADE Rating:** 

## Commentary

Peri-implantitis is a significant inflammatory condition that causes progressive bone loss around dental implants, often requiring surgical intervention when non-surgical approaches fail to restore health^1^. Among the available surgical options, guided bone regeneration (GBR) and open flap debridement (OFD) are two commonly utilized techniques. The randomized clinical trial (RCT) by Renvert et al. (2024) compared these approaches in treating peri-implantitis-related three-wall bony defects over a 36-month period^2^. The study evaluates the long-term efficacy of GBR and OFD, focusing on radiographic defect fill (RDF) and composite treatment success.

The inclusion criteria in this study, requiring three osseous walls and defects of ≥3 mm depth, aligned with the European Federation of Periodontology (EFP) guidelines^3^. This methodological decision allowed for a consistent comparison of treatment efficacy, minimizing variability due to defect characteristics. The study’s multicentre design, with calibration across centers, further contributed to the robustness of the collected data. RDF was used as the primary outcome, with GBR showing a mean RDF of 2.13 mm compared to 1.64 mm for OFD, though the difference was not statistically significant (*p* = 0.18)^2^. These findings align with previous studies, such as Isler et al.^4^, which also demonstrated the potential for GBR to improve defect fill in peri-implantitis cases.

While both GBR and OFD successfully reduced probing pocket depth (PPD) and maintained stable clinical parameters over 36 months, there were no significant differences in clinical or patient-reported outcomes (PROs)^2^. Similar satisfaction levels between the two groups suggest that GBR’s additional procedures (use of deproteinized bovine bone mineral [DBBM] and native bilayer collagen membrane [NBCM]) did not dramatically enhance patient-perceived benefits. Importantly, the use of GBR is generally recommended for cases with complex defect morphology, as its combination of materials offers potential advantages for bone regeneration. However, the possibility that some observed RDF in GBR cases includes residual graft material rather than newly regenerated bone warrants further discussion, as previously noted by Aghazadeh et al.^5^ and Schwarz et al.^6^.

Supportive peri-implant therapy was considered fundamental in maintaining the treatment results over 36 months^2^. Regular follow-up care, including professional cleaning and oral hygiene instructions, has been highlighted in several studies as required for preventing disease recurrence, especially in cases with peri-implantitis^7^. In this context, the similar long-term outcomes between GBR and OFD may partly reflect the success of the supportive care regimen rather than the specific surgical technique used. Consistent with previous literature (Aghazadeh et al.^4,8^, and Herrera et al.^3^), supportive care remains crucial in improving long-term outcomes for peri-implantitis patients.

One area of interest in this study was the use of 3% hydrogen peroxide for implant surface decontamination in both treatment groups. Hydrogen peroxide has been shown to improve healing by effectively removing microbial biofilm, a central factor in peri-implantitis pathogenesis^2,4^. The absence of adjunctive therapies such as low-level laser therapy (LLLT) or local antibiotics (e.g., doxycycline) is notable, as these interventions have been shown in other studies to improve healing outcomes^8^. The decision to omit these treatments reflects the study’s intent to focus on surgical efficacy alone, but future studies could identify the additional benefits of combining GBR or OFD with adjunctive therapies.

Another valuable consideration in evaluating this study’s findings is the disclosed financial support from Geistlich Pharma, which provided the grafting materials used in the GBR group. While there is no evidence that these relationships biased the study’s outcomes, conflicts of interest related to industry sponsorship have the potential to subtly influence study design and interpretation^9^. It is crucial to acknowledge this context when assessing the study’s conclusions, as sponsorship could impact the perceived benefits of proprietary materials, particularly in terms of treatment efficacy and long-term outcomes. Future independent studies are encouraged to validate these findings under neutral funding conditions, ensuring objectivity and reducing the risk of bias.

In summary, the study compared the treatment of peri-implantitis-related bony defects using GBR versus OFD. While GBR appeared to offer a slight advantage in defect fill and composite treatment success, the differences were not statistically significant, and patient-reported outcomes were similar between the two techniques. The study’s strengths include its strict design and consistent treatment protocols, but future research should aim to address the limitations of industry-sponsored studies and explore the long-term benefits of adjunctive therapies. Independent investigations with larger sample sizes and longer follow-up periods will be essential for confirming the results, permitting that the most effective, patient-centered treatment approaches are adopted in clinical practice.

Practice points

- GBR with DBBM and NBCM resulted in higher average defect fill and treatment success than OFD after 3 years, though the difference was not statistically significant.
- Ongoing supportive peri-implant therapy is key for maintaining the benefits of reconstructive treatments in peri-implantitis.
- No significant difference in patient-reported outcomes between GBR and OFD indicates similar patient satisfaction with both treatment methods.
